# Antioxidant Sulfated Polysaccharide from Edible Red Seaweed *Gracilaria birdiae* is an Inhibitor of Calcium Oxalate Crystal Formation

**DOI:** 10.3390/molecules25092055

**Published:** 2020-04-28

**Authors:** Leticia Castelo Branco Peroba Oliveira, Moacir Fernandes Queiroz, Gabriel Pereira Fidelis, Karoline Rachel Teodosio Melo, Rafael Barros Gomes Câmara, Monique Gabriela Chagas Faustino Alves, Leandro Silva Costa, Dárlio Inácio Alves Teixeira, Raniere Fagundes Melo-Silveira, Hugo Alexandre Oliveira Rocha

**Affiliations:** 1 Laboratório de Biotecnologia de Polímeros Naturais (BIOPOL), Departamento de Bioquímica, Centro de Biociências, Universidade Federal do Rio Grande do Norte (UFRN), 59078-970 Natal, Rio Grande do Norte-RN, Brazil; lecastelo@hotmail.com (L.C.B.P.O.); moacirfqn@gmail.com (M.F.Q.); gabrielfideliss@gmail.com (G.P.F.); melo.krt@gmail.com (K.R.T.M.); rafael_bgc@yahoo.com.br (R.B.G.C.); monique.gabi@gmail.com (M.G.C.F.A.); leandro-silva-costa@hotmail.com (L.S.C.); darlioteixeira@gmail.com (D.I.A.T.); ranierefagundes@hotmail.com (R.F.M.-S.); 2 Programa de Pós-graduação em Ciências da Saúde, Universidade Federal do Rio Grande do Norte (UFRN), 59078-970 Natal, Rio Grande do Norte-RN, Brazil; 3 Escola Multicampi de Ciências Médicas do Rio Grande do Norte, Universidade Federal do Rio Grande do Norte (UFRN), 59300-000 Caicó, Rio Grande do Norte-RN, Brazil; 4 Instituto Federal de Educação, Ciência e Tecnologia do Rio Grande do Norte (IFRN), 59190-000 Canguaretama, Rio Grande do Norte-RN, Brazil

**Keywords:** sulfated galactan, red seaweed, antioxidant, calcium oxalate dihydrate crystals, urolithiasis

## Abstract

The genus *Gracilaria* synthesizes sulfated polysaccharides (SPs). Many of these SPs, including those synthesized by the edible seaweed *Gracilaria birdiae*, have not yet been adequately investigated for their use as potential pharmaceutical compounds. Previous studies have demonstrated the immunomodulatory effects of sulfated galactans from *G. birdiae*. In this study, a galactan (GB) was extracted from *G. birdiae* and evaluated by cell proliferation and antioxidant tests. GB showed no radical hydroxyl (OH) and superoxide (O_2_^−^) scavenging ability. However, GB was able to donate electrons in two further different assays and presented iron- and copper-chelating activity. Urolithiasis affects approximately 10% of the world’s population and is strongly associated with calcium oxalate (CaOx) crystals. No efficient compound is currently available for the treatment of this disease. GB appeared to interact with and stabilize calcium oxalate dihydrate crystals, leading to the modification of their morphology, size, and surface charge. These crystals then acquired the same characteristics as those found in healthy individuals. In addition, GB showed no cytotoxic effect against human kidney cells (HEK-293). Taken together, our current findings highlight the potential application of GB as an antiurolithic agent.

## 1. Introduction

The earliest records of seaweed use as a human food source date back to the 4th century in Japan and the 6th century in China. Since then, seaweed became a food source constituting up to 25% of the human diet in countries such as Japan, China, and South Korea. Furthermore, North and South American regions, which are inhabited by Asian descendants, have also increased their seaweed consumption. In addition, seaweed consumption is also gaining popularity as a new dietary habit in Europe, mainly in France, Italy, Greece, and Ireland [1]. These marine organisms have favorable nutritional values due to their high carotenoids, proteins, dietary fiber, essential fatty acids, vitamins (C, D, E, K vitamins and B complex), and mineral contents [2]. Additionally, seaweed contains approximately 60 distinct elements, including calcium, phosphorus, sodium, magnesium, iron, copper, manganese, potassium, vanadium, and iodine [2].

Recently, our group improved the food of mice with the addition of *Gracilaria birdiae* red seaweed. After 21 days, the consumption of seaweed reduced the body weight gain and blood glucose levels in mice. In addition, it increased the Trolox equivalent antioxidant capacity, glutathione reductase, and catalase levels compared to those of the control group [3], which indicated that the oxidative stress of the animals was reduced due to the consumption of seaweed. Several papers have shown that seaweeds are also good sources of various bioactive compounds, many of which have a pharmaceutical potential [4,5]. One of these compound groups is made up of sulfated polysaccharides. The *Gracilaria* genus (red seaweed) has been previously described as the most promising marine source of polysaccharides due to its ability to produce sulfated galactans in large quantities [6]. Some species have presented commercial growth in countries that cultivate such seaweeds, including the edible *G. birdiae* in Brazil [7].

Based on infrared and nuclear magnetic resonance imaging results, the major structural features of the sulfated galactan (agaran) synthesized by *G. birdiae* is mainly composed of alternating residues of 4-linked-3,6-anhydro-α-l-galp and 3-linked-β-d-galp, with the possibility of a α-l-galp unit substituted at the 6-position by sulfate ester [8]. The in vivo anti-inflammatory effects of this sulfated galactan have also been demonstrated [9], along with its potential to prevent naproxen-induced gastrointestinal damage [10] and ameliorate trinitrobenzenesulfonic acid-induced colitis in rats [11].

Kidney stone is a common disease that affects approximately 10% of the world’s population and 60–90% of the cases are caused primarily by calcium oxalate (CaOx) crystals, the most common one being calcium oxalate monohydrate (COM) [8]. The lack of urinary inhibitors enhances the growth of the CaOx crystals and allows for crystal aggregation. These CaOx crystals are difficult to excrete and remain for a longer time in the renal system, allowing them to interact with the renal epithelium [12]. When crystals interact with renal epithelium cells, they can cause oxidative damage, which can lead to cell death, thereby damaging nearby cells; this initiates a destructive cycle: In the damaged area, crystal adhesion is easier, inducing more oxidative damage, leading to more cell death, and so on [13,14].

Despite the advancements in medical science, no adapted drugs are currently available to treat urolithiasis [15]. Therefore, ongoing research projects are focused on finding potential compounds to provide an effective treatment to/for urolithiasis. Furthermore, researchers are also searching for antioxidant compounds that can combat the oxidative stress that occurs during the formation of renal calculi [16]. It has been confirmed that sulfated polysaccharides from brown seaweed inhibit the formation of oxalate crystals, and therefore protect the renal tissue from the oxalate-related damage [17,18]. However, to our knowledge, no data has been published yet about sulfated galactans from red seaweeds.

We modified a previously described sulfated polysaccharide extraction method [8] by adding a proteolysis step to eliminate protein contamination. Through this approach, we obtained sulfated galactan from *G. birdiae* and evaluated its antioxidant and antiproliferative effects. In addition, we assessed its pharmacological potential to inhibit calcium oxalate crystal formation.

## 2. Results and Discussion

### 2.1. The Characterization of GB

After the extraction from 5 g of *G. birdiae*, we obtained 720 mg of sulfated polysaccharides, which we refer to as GB. In other words, 14% of the *G. birdiae* sample was separated into GB, which is 2.5-fold more than what was obtained by the aforementioned previous method for sulfated polysaccharide extraction from the same seaweed [8]. In seaweeds, sulfated polysaccharides are protein-bound in the cell wall. The applied extraction method directly influences the efficiency of polysaccharide extraction. The proteolytic enzyme mixture used in the extraction process promoted the degradation of carbohydrate polymer-associated proteins, which in turn enhanced polysaccharide solubilization [19]. Thus, our higher yield was due to the additional proteolysis step.

Table 1 summarizes the GB chemical analysis results. The sulfate content of the *G. birdiae*-derived polysaccharide was 10.7%, which is higher than the previously achieved 6.4% from the same seaweed [8]. GB had low protein and phenolic compound contamination (0.5% and 0.1%, respectively), which is lower than the previously described protein contamination (7.6%) [8]. On the one hand, this difference may be due to the use of proteases in the extraction method applied in this study. On the other hand, the cited previous paper did not determine the phenolic compound contamination of the presented sample, and thus we cannot compare the two results from this aspect.

The main monosaccharides identified in GB were galactose, glucose, arabinose, and xylose (Table 1). The monosaccharide composition found in GB was different from those described previously, where galactose was the major component [8]. To our knowledge, monosaccharide composition changes of sulfated polysaccharides from the same red seaweed have not yet been reported. However, the sulfated polysaccharides produced by other seaweeds (brown and green) show variability, depending on the place and season of collection. For instance, sulfated galactofucans extracted from the brown seaweed *Saccharina longicruris* at four different collection periods (May, August, and November 2005 and June 2006) showed different monosaccharide composition [20]. Therefore, apart from the distinct extraction methods, the differences in locality and seasonality greatly influence seaweed biotic and abiotic factor composition, which would also explain the differences in their monosaccharide composition.

### 2.2. GB Antioxidant Effect Evaluation

The substrate oxidation process consists of three stages (initiation, propagation, and termination). Antioxidants can act at any of these steps, and the more steps a compound intervenes at, the better is the antioxidant. Sulfated polysaccharides extracted from seaweed have already been identified as antioxidants [21]. Polysaccharides from *G. birdiae* also have antioxidant properties and have been evaluated by a DPPH scavenging test [22]. Several in vitro antioxidant tests are available to assess the antioxidant activity of biomolecules [23]. In the current study, we used six methods to evaluate the possible effects of GB on the initiation (total antioxidant capacity and reducing power), propagation (iron and copper chelation), and termination (superoxide and hydroxyl radical scavenging activities) steps.

Initially, GB was analyzed using the total antioxidant capacity (TAC) test. GB had activity equivalent to 86.6 ± 10.8 mg of ascorbic acid, which is greater than those of sulfated polysaccharides extracted from the seaweeds *Sargassum filipendula* [24], *Dictyopteris justii* [17], and *G. caudata* [18]. Therefore, GB can be considered as a good electron donor.

**Figure 1:**
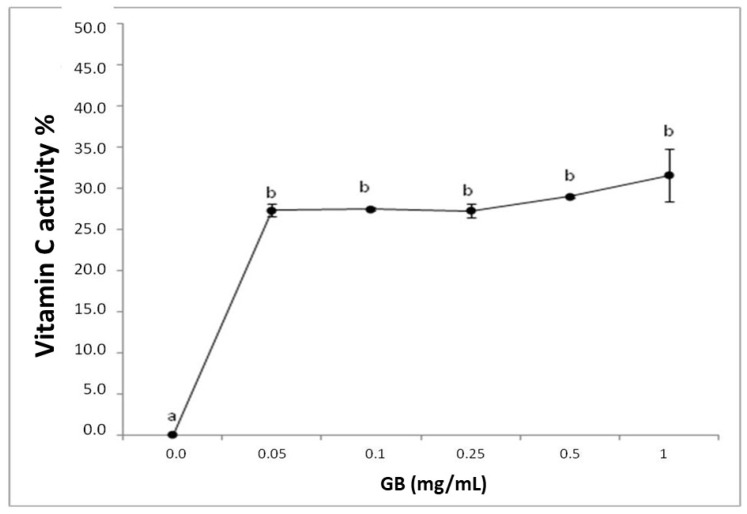
Representation of the reducing power of *G. birdiae* GB. Data are expressed as mean ± standard deviation. Reducing power is expressed as a percentage of the activity of 0.2 mg/mL of vitamin C. The letters “a and b” indicate significant differences between the concentrations of sulfated polysaccharides obtained using one-way ANOVA, followed by the Student-Newman-Keuls test (*p* < 0.05).

The reducing power test is expressed with the reductive activity equivalent and is calculated from the ratio of the sample’s reductive activity and that of 0.2 mg/mL vitamin C. GB had a reducing effect, however, it was not dose-dependent (Figure 1).

Both the TAC and the reducing power tests assess the ability of a sample to donate electrons in different chemical environments. Therefore, GB can be considered a good electron donor in different environments, suggesting that GB may donate electrons and act as an antioxidant at different cellular sites, such as the mitochondria, cytoplasm, and nucleus.

GB has not yet been described as a hydroxyl or superoxide radical scavenger. Polysaccharides from other seaweeds have been reported to have low or no ability to scavenge these radicals [19], indicating that the primary antioxidant mechanism of seaweed sulfated polysaccharides is likely different.

**Figure 2:**
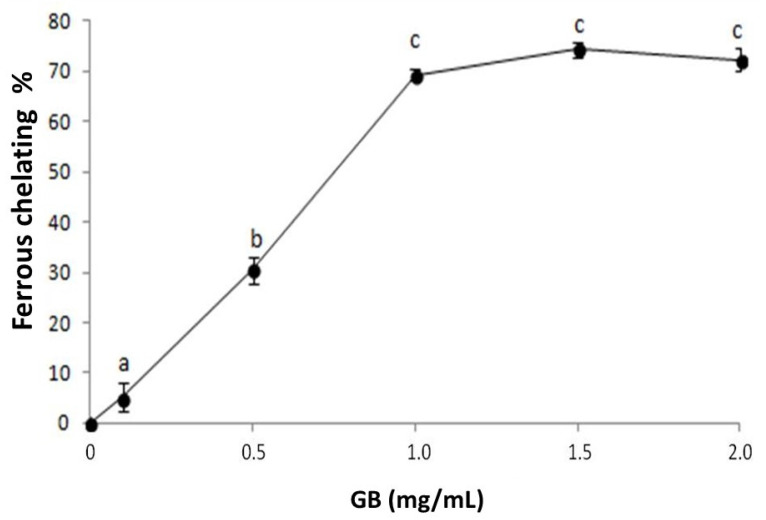
GB chelating effect on ferrous ions. Data are expressed as mean ± standard deviation. The letters “a–c” indicate significant differences between GB concentrations obtained using one-way ANOVA, followed by the Student-Newman-Keuls test (*p* < 0.05).

We investigated the iron chelation activity of GB (Figure 2). GB has a dose-dependent effect that stabilizes around 1 mg/mL, showing 69.3% ± 1.1 iron-chelation activity. This activity is greater than that observed in other seaweed sulfated polysaccharides [25,26] and is similar to those of the sulfated polysaccharides from *G. caudata* [19] and *Caulerpa cupressoides* [27]. These results show that GB is a good iron chelator and can act as an antioxidant by inhibiting the Fenton reaction, in which iron reacts with hydrogen peroxide and produces a hydroxyl radical, one of the most damaging radicals [24].

**Figure 3:**
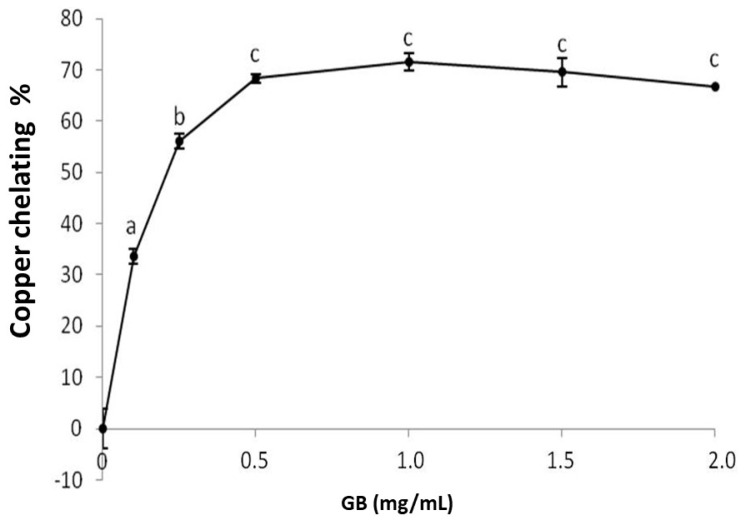
Copper chelating activity of GB. Data are expressed as mean ± standard deviation for three experiments. The letters “a–c” indicate a significant difference (*p* < 0.05) between concentrations obtained using one-way ANOVA, followed by the Student-Newman-Keuls test (*p* < 0.05).

GB chelates copper in a dose-dependent manner, stabilizing around 1 mg/mL, while the chelation index approaches 71% (Figure 3). Sulfated polysaccharides, specifically sulfated fucans extracted from the seaweed *D. justii*, have been previously shown to chelate copper with a copper-chelation activity of 65% (2 mg/mL). The sulfated glucans of the same algae had a copper chelation activity around 80% (2 mg/mL) [17], which is higher than that of GB.

The copper concentration increase is followed by an increased production of reactive oxygen species due to the Fenton [27] and Haber-Weiss [28] reactions. In addition, the preformed lipid hydroperoxides (LOOH) are decomposed through the Fenton reaction to form alkoxy radicals (LO), strong oxidizers that propagate the lipid peroxidation chain reaction or react with other cellular constituents. Therefore, Cu^2+^ chelation might be crucial to the prevention of the production of reactive species that damage biomolecules. Therefore, the copper-chelating ability of GB confirms its antioxidant potential.

### 2.3. In Vitro Assay for Inhibition of Calcium Oxalate Crystallization

The antioxidant properties of GB might be important for treating several diseases, such as cancer and atherosclerosis, and in the protection of tissues against oxidative damage. For instance, antioxidants, such as vitamin E and ascorbic acid, protect kidney tissue from damage caused by reactive oxygen species (ROS) resulting from high concentrations of extra- and intracellular calcium oxalate [29]. For this reason, researchers are looking for antioxidant compounds that can combat oxidative stress that occurs during the formation of kidney stones [16]. Sulfated polysaccharides from the brown algae *Sargassum graminifolium* can reportedly inhibit calcium oxalate crystals formation in vitro [30]. The ability of red algae polysaccharides to inhibit oxalate crystal formation has not yet been investigated. As GB has antioxidant activity, we investigated whether it could inhibit oxalate crystal formation and/or growth in vitro.

Calcium oxalate must reach the supersaturation level and exceed the meta stabile limit in the urine for crystal formation. This results in the formation of the crystal nucleus, which consists of calcium oxalate ions (nucleation). The crystal core grows in an orderly manner (growth) with the incorporation of additional ions, generating a nanocrystal. The growing nanocrystals aggregate and form clusters (aggregation) that precipitate [31].

Our results show that GB’s (125 mg) increased nucleation by 6.2%. We observed that increasing GB amounts (250 mg) raised the level of nucleation stimulation to 67.2%. In contrast to our results, many reports demonstrate that oxalate crystal nucleation could be inhibited in the presence of polyanions, such as proteins and polysaccharides [32]. However, it has also been shown that a sulfated glucan from the seaweed *D. justii* does not inhibit crystal nucleation, indicating that the position of the sulfate groups along the polysaccharide chair affects this nucleation much more than charge density or sulfate content [17]. We observed that GB inhibited aggregation at 15.2% (125 mg) and 28.2% (250 mg). These results are lower compared to those obtained with sulfated polysaccharides from *S. graminifolium*, which inhibited nucleation by 76% [31].

Taken together, GB stimulates crystal formation but inhibits aggregation, possibly by preventing the formation of large crystals. To confirm this hypothesis, we verified the size and morphology of CaOx crystals formed in the presence of GB.

CaOx crystals can be monohydrated (COM), di-hydrated (COD), or tri-hydrated (COT). COM crystals have an elongated tetragonal geometry, with an irregular outer surface, a dense structure, and high hardness prism. The COM calcifications consist of a core, where the crystals are deposited concentrically, and a middle tier that is radially striated. COD crystals have a bipyramid tetragonal geometry and are thermodynamically unstable. Upon contact with liquid, COD crystals gradually transition into the more stable COM form. Large amounts of the COM form are found in kidney stones, while the COD form is rare. COT is thermodynamically unstable and is rarely found in calcifications [33,34].

**Figure 4:**
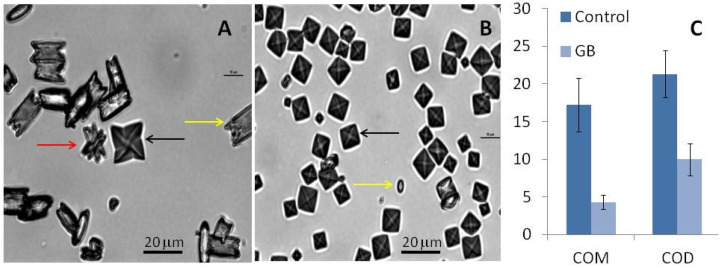
CaOx crystal micrographs obtained by inversion microscopy. Crystals were formed in the metastable solution of CaOx in the absence (**A**) and presence (**B**) of GB (0.1 mg/mL). Yellow arrows indicate monohydrated (COM) crystals, black arrows indicate di-hydrated (COD) crystals, and red arrow indicate tri-hydrated (COT) crystals. Average size of formed crystals (**C**). Scale bar represents 20 μm.

Under control conditions, all three types of CaOx crystals can be formed (Figure 4). In the absence of GB, the average number of crystals per field was 14 ± 0.7 while in its presence, the number of crystals increased to 47 ± 6 per field. These results confirm that GB stimulates oxalate crystal formation. Moreover, we observed that the average ratio of COD:COM was 1:6 per field under control conditions. In the presence of GB, the same ratio was 3:1 per field.

Furthermore, we observed that the size and morphology of these crystals also changed in the presence of GB. The COM crystals in the control group had a rectangular morphology (Figure 4A) and an average size of 17.2 ± 3.5 µm (Figure 4C). In the presence of GB, the COM crystals had an imperfect rectangular morphology (Figure 4B) and their average size was 4.3 ± 0.9 µm (Figure 4C). GB also affected the size of the COD crystals: The size of these crystals decreased from 21.3 ± 3.0 µm to 9.98 ± 2.1 µm in the presence of GB. The reduction of crystal size in the presence of GB confirms the aggregation-inhibition results. GB inhibits aggregation, which results in smaller crystals.

**Figure 5:**
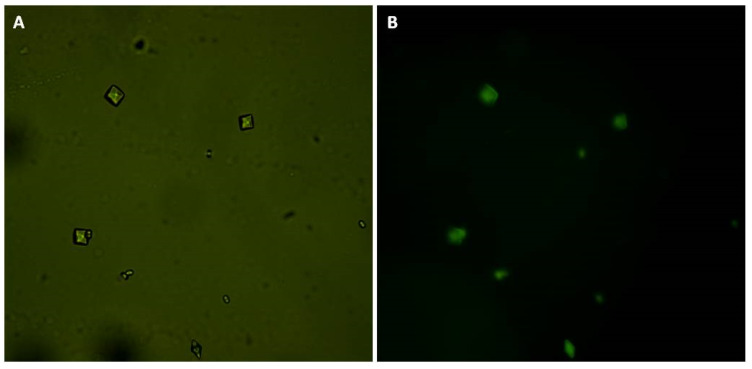
CaOx crystals formed in the presence of GB-FITC. (**A**) CaOx crystals observed under white light and (**B**) using fluorescent microscopy.

Regarding the crystal morphology, in the presence of GB, more spheroidal COM and COD crystals could be observed (Figure 5B), which indicates that GB interferes with the crystalline network and prevents optimal CaOx crystals formation.

The increase in the number of formed crystals indicates that GB stimulates nucleation, while the decreasing size of the crystals shows that it also inhibits aggregation. We hypothesize that GB, when interacting with calcium oxalate, functions as a nucleus for crystal formation. When more GB molecules are present, more crystals are formed. With many nuclei growing at the same time, the calcium oxalate is consumed, the concentration falls below the saturation level, and the crystals stop growing. Hence, GB results in smaller crystals and aggregation inhibition.

We can also see that GB affects crystal morphology. A more rounded COM and COD crystal geometry indicates they are more amorphous, which is caused by the disrupted crystalline network due to the presence of GB. This geometry has less surface area compared to crystals with edges and tips, as are formed in the absence of GB.

Despite its instability, the COD crystal is common in healthy urine. This indicates that urine naturally has molecules that stabilize the COD form and prevent its transformation into the geometric COM form. Thus, we suggest that GB acts as a COD crystal stabilizer and could be used in urolithiasis treatment.

Next, we investigated the interaction between GB and the crystals by measuring the zeta (ζ) potential in the presence and absence of GB. The ζ potential measures the total particle surface charge (including molecules) relative to the liquid suspension in which the particles are suspended. The crystals had a positive ζ potential (9.76 ± 0.16) in the absence of GB, which is due to their positively charged calcium ions. However, in the presence of GB, the crystals had a negative ζ potential (−23.42 ± 0.13). As GB is sulfated, which is negatively charged under the crystal-formation pH conditions, the negative ζ potential is the result of the presence of GB.

Finally, we bound GB to FITC. Subsequently, we induced crystal formation in the presence of GB-FITC particles. The formed crystals were then visualized using fluorescence microscopy. GB-FITC was associated to the crystals (Figure 5). These results indicate that GB associates with formed crystals, including the crystal surface, which allows COD crystal stabilization.

### 2.4. Evaluation of GB Cytotoxicity against Human Renal Cells

**Figure 6:**
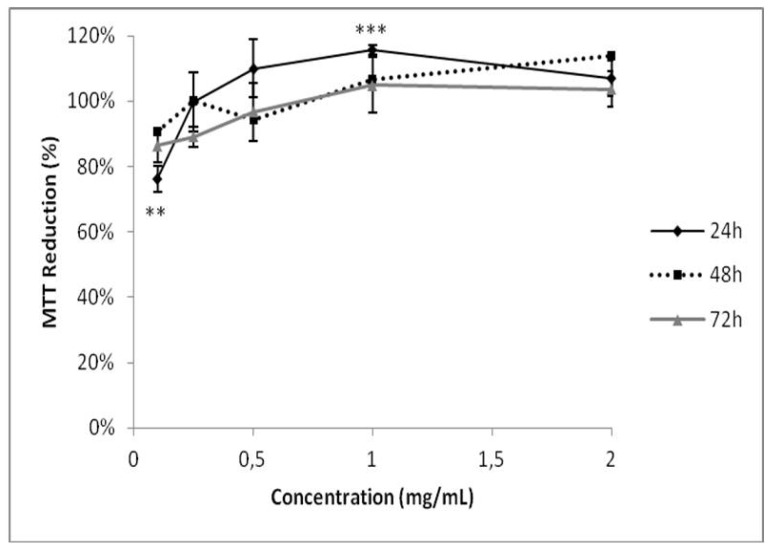
Evaluation of GB toxicity on HEK-293 cell viability. The cells were grown in a range of GB concentrations (0.125–2.000 mg/mL) for 24, 48, or 72 h. Cell viability was measured using the MTT test. ** (*p* < 0.05) and *** (*p* < 0.001) indicate significant differences between the concentrations obtained using one-way ANOVA, followed by the Student-Newman-Keuls test.

As GB participates in the formation of calcium oxalate crystals, we investigated its possible cytotoxicity in HEK-293 kidney cells. GB showed no cytotoxicity under any evaluated conditions (Figure 6). These data are consistent with previous publications showing that polysaccharides from six different seaweeds did not show any cytotoxic effect in renal cells [35]. GB has antioxidant activity, inhibits the growth and formation of COM crystals, and has no cytotoxic effect against human renal cells, these data suggest that GB could be used as a possible compound in the treatment of urolithiasis, following further experimental confirmation. As *G. birdiae* is edible, it could be used as a functional food with antioxidant activity in treating urolithiasis. Therefore, seaweed toxicity assays are required both in animals and humans to determine safe levels of its daily consumption.

## 3. Materials and Methods

### 3.1. Materials

Potassium ferricianyde, ferrous sulfate II, trichloroacetic acid, and sulfuric acid were purchased from Merck (Darmstadt, Germany). Nitro Blue Tetrazolium (NBT), monosaccharides, diaminoethanetetraacetic acid (EDTA), ascorbic acid, methionine, 3-(4,5-dimethylthiazolyl-2)-2,5-diphenyltetrazolium bromide (MTT), Griess reagent, pyrocatechol violet, riboflavin, and ammonium molybdate were purchased from Sigma-Aldrich Co. (St. Louis, MO, USA). Sodium bicarbonate, non-essential amino acids, and phosphate buffered saline (PBS) were purchased from Invitrogen Corporation (Burlington, ON, Canada). Dulbecco′s modified Eagle′s medium (DMEM) and fetal bovine serum (FBS) were obtained from CULTILAB (Campinas, SP, Brazil). Penicillin and streptomycin were obtained from Gibco (Fort Worth, TX, USA). All other solvents and chemicals were of analytical grade.

The HEK-293 cells (ATCC^®^ CRL-1573™) were grown in a DMEM medium with 10% FBS, 100 μg/mL streptomycin and 100 IU/mL penicillin. The cells were cultured in DMEM supplemented with FBS (10% *v*/*v*) and antibiotics (100 U/mL penicillin and 100 μg/mL streptomycin), at 37 °C in a humidified atmosphere with 5% CO_2_. For maintenance of the cells, the culture medium was changed every three days, and the cells were further subcultured using trypsin/EDTA purchased from CULTILAB (Campinas, SP, Brazil).

### 3.2. Sulfated Galactan Extraction from Gracilaria birdiae

The mature seaweeds (35–40 cm long) were collected at Rio do Fogo Beach (Rio Grande do Norte, Brazil—5°16′16″ S/35°22′54″ W) by local fishermen in October 2016. They were cleaned to eliminate residues, encrusted organisms, and epiphytes and taken to the laboratory (Laboratório de Biotecnologia de Polímeros Naturais, Departamento de Bioquímica, Universidade Federal do Rio Grande do Norte, RN). The seaweed was then identified based on its morphology [36]. The seaweed has an erect thallus growing up to 40 cm in height and up to 2.3 mm in width. Its color varies between light red and dark red. It is terete throughout and usually branches to two or four (less common) orders. The branching is subdichotomous to unilateral, and there is either a single main axis or several, arising from a discoid holdfast. The specimens showed prominent cystocarps (from 0.9 to 1.2 mm in diameter and ~1.0 mm high) scattered over the thallus. The material collection occurred under the authorization of the Brazilian National System of Management of Genetic Heritage and Associated Traditional Knowledge SISGEN n° A72AD2B.

The dry seaweed was then powdered and treated with 2 volumes of ethanol (99.5%, Sigma-Aldrich Co., St. Louis, MO, USA) overnight 5 times to reduce the amount of pigments in the sample, as previously described [37]. The supernatant was eliminated, and the power was dried at 50 °C under ventilation. The dilapidated dried material was then packed in polyethylene bags and stored at room temperature in the dark.

The GB extraction was performed as previously described [8] with modifications. Approximately 5 g of powdered alga was suspended with four volumes of 0.25 M NaCl and the pH was adjusted to 8.0 with NaOH. Next, 75 mg of Prolav 750 alkaline protease mixture (Prozyn Biosolutions, São Paulo, SP, Brazil) was added for proteolytic digestion and the solution was incubated for 24 h at 60 °C under agitation and periodical pH adjustments. The mixture was then filtered through a cheesecloth and the obtained soluble GB was precipitated with two volumes of ice-cold methanol. After 24 h, the GB was collected using centrifugation (10,000× *g*, 20 min), vacuum dried, suspended in distilled water, and analyzed.

### 3.3. Chemical Analysis and Monosaccharide Composition

GB was submitted to acid hydrolysis (4 M HCl, 100 °C, 6 h) and its sulfate content was determined using the gelatin-barium method [38] using sodium sulfate as the standard. The protein content was measured using the Spector’s method [39]. Total sugar was estimated by the phenol-H_2_SO_4_ reaction [40] using D-galactose as the standard. Total phenolic compounds were quantified by the colorimetric technique of Folin-Ciocalteu as described earlier [24], applying gallic acid as the standard.

To determine the best polysaccharide acid hydrolysis using HCl, that is, where polymer degradation occurs without destroying monosaccharides released by this degradation, GB was hydrolyzed with 0.5 M, 1 M, 2 M, and 4 M HCl for 30 min, 1 h, 2 h, and 4 h, respectively. A temperature of 100 °C was maintained in all hydrolysis conditions. The material was later neutralized, dried, and resuspended in water, and reducing sugars were determined by the Somogyi-Nelson method [41]. The best hydrolysis condition was 2 M of HCl for 2 h. Thus, GB was hydrolyzed (2 M HCl, 100 °C, 2 h) and its sugar composition was determined using a LaChrom Elite^®^ HPLC system from VWR-Hitachi with a refractive index detector (RI detector model L-2490). A LichroCART^®^ 250-4 column (250 mm × 40 mm) packed with Lichrospher^®^ 100 NH2 (5 µm) was coupled to the system. The column was eluted with acetonitrile, gradient-grade, for liquid chromatography (Merck, Darmstadt, Germany) and ultrapure water (80:20 *v*/*v*). The used sample mass was 0.2 mg and the analysis time was 25 min. The following sugars were analyzed as references: arabinose, fructose, fucose, galactose, glucose, glucosamine, glucuronic acid, mannose, and xylose.

### 3.4. Antioxidant Activity

Six assays were performed to analyze the GB antioxidant activity as described earlier [17,24]: total antioxidant capacity (TAC), reducing power, hydroxyl radical scavenging, superoxide radical scavenging, and cupric and ferric chelating.

#### 3.4.1. Determination of Total Antioxidant Capacity

This assay is based on the reduction of Mo (VI) to Mo (V) by GB and subsequent formation of a green phosphate/Mo (V) complex at an acidic pH. Tubes containing GB and the reagent solution (0.6 M sulfuric acid, 28 mM sodium phosphate, and 4 mM ammonium molybdate) were incubated at 95 °C for 90 min. After the incubation, the mixture was cooled to room temperature and the absorbance of each solution was measured at 695 nm against a blank. The total antioxidant capacity was expressed as vitamin C-equivalent.

#### 3.4.2. Hydroxyl Radical Scavenging Activity Assay

The scavenging activity of GB against the hydroxyl radical was investigated using the Fenton’s reaction (Fe^2+^ + H_2_O_2_ → Fe^3+^ + OH^−^ + OH). These results were expressed as an inhibition rate. Hydroxyl radicals were generated using 3 mL sodium phosphate buffer (150 mM, pH 7.4), which contained 10 mM FeSO_4_·7H_2_O, 10 mM EDTA, 2 mM sodium salicylate, 30% H_2_O_2_ (200 mL), and varying polysaccharide concentrations. In the control, sodium phosphate buffer replaced H_2_O_2_. The solutions were incubated at 37 °C for 1 h, and the presence of the hydroxyl radical was detected by monitoring absorbance at 510 nm. Gallic acid was used as a positive control.

#### 3.4.3. Superoxide Radical Scavenging Activity Assay

This assay was based on the capacity of GB to inhibit the photochemical reduction of nitro blue tetrazolium (NBT) in the riboflavin-light-NBT system. Each 3 mL of reaction mixture contained 50 mM phosphate buffer (pH 7.8), 13 mM methionine, 2 mM riboflavin, 100 mM EDTA, NBT (75 mM), and 1 mL sample solution. After the production of blue formazan, the absorbance increase was determined at 560 nm after 10 min illumination from a fluorescent lamp. The entire reaction assembly was enclosed in a box lined with aluminum foil. Identical tubes with the reaction mixture were kept in the dark and served as blanks. Gallic acid was used as a positive control.

#### 3.4.4. Ferric Chelating

The ferrous-ion-chelating ability of the samples was investigated using the following methodology: Sulfated polysaccharides at different concentrations were introduced to the reaction mixture, which contained FeCl_2_ (0.05 mL, 2 mM) and ferrozine (0.2 mL, 5 mM). The mixture was shaken and incubated for 10 min at room temperature and the absorbance of the mixture was measured at 562 nm against a blank. EDTA was used as a positive control.

#### 3.4.5. Copper Chelation

To determine the ability of GB to chelate copper ions we used a previously described method [17]. Pyrocatechol Violet associates with cations, including aluminum, copper, bismuth, and thorium. In the presence of chelating agents, this association is not formed, resulting in a less intense color change. Different concentrations of the samples (0.1–2.0 mg/mL), Pyrocatechol Violet (4 mM), and copper II sulfate pentahydrate (50 µg/mL) were mixed in a 96-well plate and the absorbance was measured at 632 nm. EDTA was used as a positive control.

#### 3.4.6. Reducing Power

The reducing power was quantified as described previously [17]. Different concentrations (0.1–1.0 mg/mL) of GB (4 mL) were mixed with a phosphate buffer (0.2 M, pH 6.6) containing potassium ferricyanide (1%) and incubated for 20 min at 50 °C. The reaction was stopped by the addition of TCA (trichloroacetic acid) to 10%. Subsequently, distilled water and ferric chloride (0.1%) were added to the samples. Readings were taken at 700 nm.

### 3.5. Calcium Oxalate Crystallization Test

We measured the effect of GB on calcium oxalate crystallization using a spectrophotometer for 30 min at 620 nm as previously described [31]. In summary, the calcium chloride solution (1.0 mL) containing different concentrations of sulfated polysaccharides or sodium citrate (a positive control) was stirred constantly at 37 °C. After obtaining a stable baseline, crystallization was induced by adding the sodium oxalate solution (1.0 mL), resulting in final concentrations of 4 mM calcium and 0.5 mM oxalate. From a linear regression analysis, we measured the rate of crystallization inhibition. The referring percentage was calculated from the rate of nucleation and aggregation as follows: [1 − (SNA/SNC)] × 100 for the percentage of nucleation, having SNA as the inclination of the absorbance of the salt solution in the presence of the samples and NSC is the inclination of the control; [1 − (SAA/SAC)] × 100 for the percentage of aggregation where SAA is the inclination of the absorbance of the solution in the salts in the presence of samples and SAC is the inclination of the absorbance of the control.

### 3.6. Crystal Morphology Image Analysis

Crystal formation was induced in the presence or absence of GB. After 30 min, the solutions were centrifuged (10 min., 5000× *g*) and the supernatant was discarded. The crystals were suspended in 0.5 mL of water, 0.1 mL was put in a micrograph and analyzed by bright field microscope (Nikon Eclypse Ti-E, Nikon Co., Tokyo, Japan). The crystal morphology was analyzed in ten randomly selected fields at 60× magnification. Three different experiments were performed.

### 3.7. Measure of Zeta Potential (ζ)

Crystal formation was induced in the presence or absence of GB. After 30 min, the solutions were centrifuged (10 min., 5000× *g*) and the supernatant was discarded. The crystals were suspended in 1.5 mL of water and the zeta potential (ζ) of the samples was obtained using the Zeta Plus Analyzer NanoBrook 173 (Brookhaven Instruments, Holtsville, NY, USA).

### 3.8. Conjugation of GB with Fluorescein

First, 5 mg of GB was mixed with 1 mg of fluorescein (Sigma-Aldrich Co.; St. Louis, MO, USA) in a 0.1 M solution of PBS (pH 7.0) for 1 h in the dark at room temperature. The solution was dialyzed through a MWCO 3.5 kDa membrane tube and lyophilized at the end. GB-FITC was used in the calcium oxalate crystallization test as described above.

### 3.9. MTT [3-(4,5-dimethylthiazol-2-yl)-2,5-diphenyltetrazolium Bromide] Assay

The MTT assay was performed as described previously [42]. HEK-293 cells were plated onto 96-well plates (5 × 10^3^ cells/well) and incubated overnight at 37 °C in 5% CO_2_. After 24 h incubation, the cells war rinsed with PBS and the medium was replaced by a DMEM without FBS, followed by incubation for another 24 h in order to stimulate cells to enter in G0 phase. The medium was replaced by a DMEM with 10% FBS with GB (125, 250, 500, 1000, 1500, or 2000 mg/mL) and the cells were incubated for 24, 48, or 72 h. The remaining GB was rinsed with PBS. Then, 100 µL of MTT (12 mM, final concentration) was dissolved in PBS and added to determine the effect of GB on cell proliferation. The cells were incubated for 4 h at 37 °C in 5% CO_2_. To solubilize the MTT-reduced product, 100 µL isopropanol containing 0.04 N HCl was added to each well and thoroughly mixed. After 15 min, the absorbance at 570 nm was read using a Multiskan Ascent plate reader (Thermo Labsystems, Franklin, MA, USA). The percentage of cell proliferation was calculated as follows:

Cell proliferation % = (Absorbance of sample)/(Absorbance of the control) × 100(1)

### 3.10. Statistical Analysis

All data are expressed as average ± standard deviation. Statistical analysis was performed using one-way ANOVA. Student-Newman-Keuls post-tests were carried out for multiple group comparisons. In all cases, *p* < 0.05 was considered statistically significant. Statistical analysis was performed using GraphPad Prism 5.01 (GraphPad Software Inc., La Jolla, CA, USA).

## 4. Conclusions

In this study, we purified GB, a sulfated galactan, from the red seaweed *G. birdiae.* GB did not present significant hydroxyl or superoxide radical scavenging activity, but it showed relevant antioxidant activity in four different tests. In addition, GB inhibited aggregation and modified the morphology of calcium oxalate crystals. Furthermore, GB was found to be non-cytotoxic to human kidney cells. Taken together, our current findings highlight the potential application of GB as an antiurolithic agent. Further in vivo studies are necessary to verify our in vitro data and to further examine the possible use of GB in treating and preventing urolithiasis.

## Figures and Tables

**Table 1:** Chemical composition of sulfated polysaccharides from *G. birdiae* (which we refer to as GB).

Sample	Sugar (%)	Sulfate (%)	Protein (%)	Phenolic (%)	Molar Ratio			
					Gal	Glc	Ara	Xyl
GB	80.1 ± 0.4	10.7 ± 0.6	0.5 ± 0.05	0.1 ± 0.02	1.0	0.3	0.5	1.9

Gal—galactose; Xyl—xylose; Ara—arabinose; Glc—glucose.
